# Altered Salivary Microbiome in the Early Stage of HIV Infections among Young Chinese Men Who Have Sex with Men (MSM)

**DOI:** 10.3390/pathogens9110960

**Published:** 2020-11-19

**Authors:** Jin Li, Shenghua Chang, Haiying Guo, Yaoting Ji, Han Jiang, Lianguo Ruan, Minquan Du

**Affiliations:** 1 The State Key Laboratory Breeding Base of Basic Science of Stomatology (Hubei-MOST) & Key Laboratory of Oral Biomedicine Ministry of Education, School & Hospital of Stomatology, Wuhan University, Wuhan 430079, China; lijin891212@whu.edu.cn (J.L.); changshh2017@whu.edu.cn (S.C.); haiyingguo@whu.edu.cn (H.G.); yaotingji@whu.edu.cn (Y.J.); jianghan@whu.edu.cn (H.J.); 2 Department of Infectious Diseases, Jin Yin-tan Hospital, Wuhan 430023, China

**Keywords:** saliva, microbiome, human immunodeficiency virus (HIV), men who have sex with men (MSM), antiretroviral therapy (ART)

## Abstract

Human immunodeficiency virus (HIV) infections are spiking in Chinese young men who have sex with men (MSM). To explore alterations in the salivary microbiome and its correlation with demographic characteristics, CD4+ T cell count and viral load (VL) in HIV infections, samples of unstimulated whole saliva were analyzed by 16S rRNA gene sequencing using the Illumina MiSeq platform in 20 HIV newly infected patients before the initiation of antiretroviral therapy (ART) and at three and six months after, and in 20 age- and gender-paired healthy Chinese people. The results showed that the alpha diversity of salivary microbiota in HIV infections did not show differences from the healthy controls, but was reduced after six months under ART treatment. Comparative analysis revealed that *Streptococcus* was enriched in HIV-infected individuals, while *Neisseria* was enriched in the healthy control group. After effective ART, the salivary microbiota composition was not completely restored, although some microbiota recovered. In addition, we found *Provotella_7*, *Neisseria* and *Haemophilus* were correlated negatively with CD4+ T cell count, while *Neisseria* was correlated positively with VL. We conclude that HIV infections experience a dysbiosis of the salivary microbiome. The salivary microbiome test could be a substitute for the blood tests in the diagnosis and prognosis of diseases.

## 1. Introduction

HIV continues to be a major global public health issue. At the end of 2019, the World Health Organization (WHO) reported that there were nearly 38 million HIV-infected patients, of which 1.7 million newly infected patients were diagnosed and 690,000 people died from HIV-related diseases in 2019 globally [1]. HIV infections have surged among young Chinese MSM, and the proportion of MSM living with HIV has risen sharply according to a new survey of the risk factors assessment of HIV/acquired immunodeficiency syndrome (AIDS) among different groups of Chinese people [2]. Generally, as a result of immune impairment induced by HIV, opportunistic oral infections (oral candidiasis, oral hairy leukoplakia, linear gingival erythema, etc.) are present in 30–80% of patients living with HIV [3,4]. At the same time, as one of the earliest symptoms of AIDS, opportunistic oral infections can be an indicator for the dentists to suspect HIV infection clinically [5]. Diverse studies have revealed that after infection with HIV, both the immune system and the saliva microbiome experience specific alterations that, in turn, exacerbate oral infections [6]. There is no cure for HIV infection; however, early effective ART can inhibit virus replication dramatically and help prevent onward transmission to other people, which eventually contributes to a global reduction of the HIV-associated oral lesions [7].

Currently, diagnosis of HIV infection is performed by the detection of HIV antibodies in the blood, and the CD4+ T cell count and viral load (VL) are important parameters for the measurement of immune function, the guidance of clinical medication, the determination of disease progression, and the evaluation of the curative effect [8]. Recent advances in rapid high throughput sequencing techniques have greatly improved the exploration of nonculturable microbes, including bacteria, virus and fungi, as well as their association with our health and disease [9,10,11]. After infection with HIV, the commensal oral [12], lung [13], gut [14] and vaginal [15] microbiomes go through varying degrees of change, and some particular microbes are associated with HIV progression as well. However, both the blood test and the sampling of the microbiomes in most parts of the body sites require invasive procedures that can be traumatic and inconvenient, given the vulnerability of HIV patients to infection. Thus, the salivary microbiome is a considerably attractive alternative to explore novel biomarkers.

Saliva is a diversified biological oral fluid that has nearly 50% similarity to blood, with an abundance of human oral microbes, deoxyribonucleic acid (DNA), ribonucleic acid (RNA), proteins, metabolites, and other biological information, that this makes it a promising surrogate indicator for oral and systemic physiological and pathological conditions [16,17]. As a diagnostic sample, saliva can often be superior to serum and samples from other body sites because of the noninvasive collection method, the cost-effective access to screening large populations, and the minimal risk of infection, especially for some contagious diseases [18]. Increasing attention has been paid to salivary biomarkers for some oral diseases such as dental caries [19], periodontal diseases [20], oral squamous cell carcinoma [21], as well as for systemic diseases such as autoimmune disease (arthritis) [22], cardiovascular disease [22] and HIV [23]. Thus, saliva could be a highly effective substitute for blood in the diagnosis and prognosis of diseases, and this needs to be further explored.

Alterations in the oral microbiome are linked with HIV infection or ART [23]. Notably, there is a higher or lower abundance of putative oral microbes in HIV infections; for instance, *Streptococcus* spp. has been reported to be increased in some studies and decreased in others [12,24,25,26]. Although the oral mycobiome and virome have been less well understood, increased *Candida* spp. and oral HPV (human papillomavirus) with HIV infections have been explored [27,28]. Nonetheless, all of these studies were focused on the alterations rather than the relationship with the blood parameters in the early stage of the infection. Moreover, the sample sizes, variations in the techniques and the lack of longitudinal studies have certain limitations. Thus, we felt that further analyses of the salivary microbiome and its correlation with CD4+ T cell count and VL in HIV infections were demanded.

In the current study we aimed to investigate the impact of HIV infection and ART on the salivary microbiome, and the possible relationship of the salivary microbiome with the CD4+ T cell count and the VL in serum. We utilized bacterial 16sRNA next-generation sequencing to characterize the salivary microbiome in 20 HIV-uninfected controls versus 20 HIV-infected patients, and a longitudinal study was applied to explore the salivary microbiome in the HIV-infected group at three and six months after the initiation of the ART. We found that the salivary microbiomes of HIV-infected patients and healthy controls differed significantly. After effective ART, the salivary microbiota composition can be partially reversed. In addition, we found that *Provotella_7*, *Neisseria* and *Haemophilus* were correlated negatively with CD4+ T cell count, while *Neisseria* was correlated positively with VL. The salivary microbiome test could be a substitute for blood tests in the diagnosis and prognosis of diseases.

## 2. Results

### 2.1. Study Cohort

Twenty HIV-infected young Chinese men newly diagnosed within one year and before ART were enrolled in our study. All patients were men aged 26.30 ± 5.41 years (range: 20–36), and all of them were MSM. The baseline CD4+ T cell count was 365.85 ± 111.88 cells/μL (range: 219–652) and the HIV-1 RNA loads were 580,006.45 ± 1,903,549.52 copies/mL in blood (range: 5059–8,749,628). After ART for six months, the CD4+ T cell count increased and VL decreased (Table 1).

### 2.2. Salivary Microbiome Diversity between Healthy Controls and HIV Infections over the Course of ART

**Figure 1:**
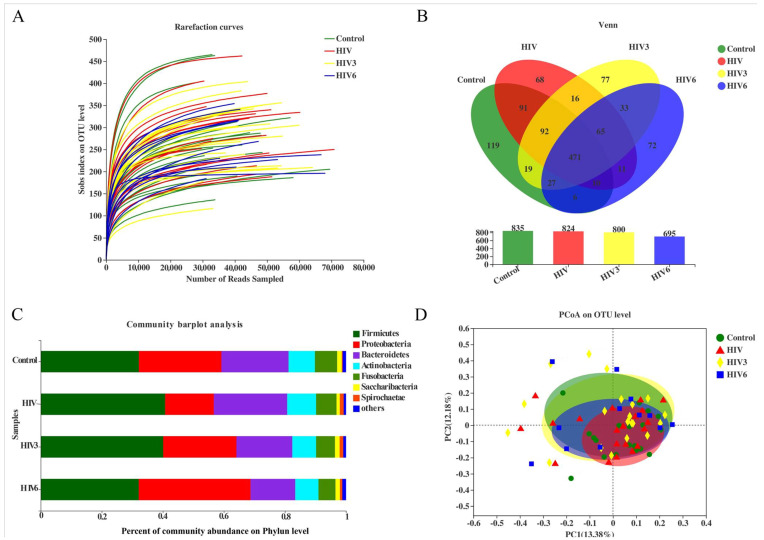

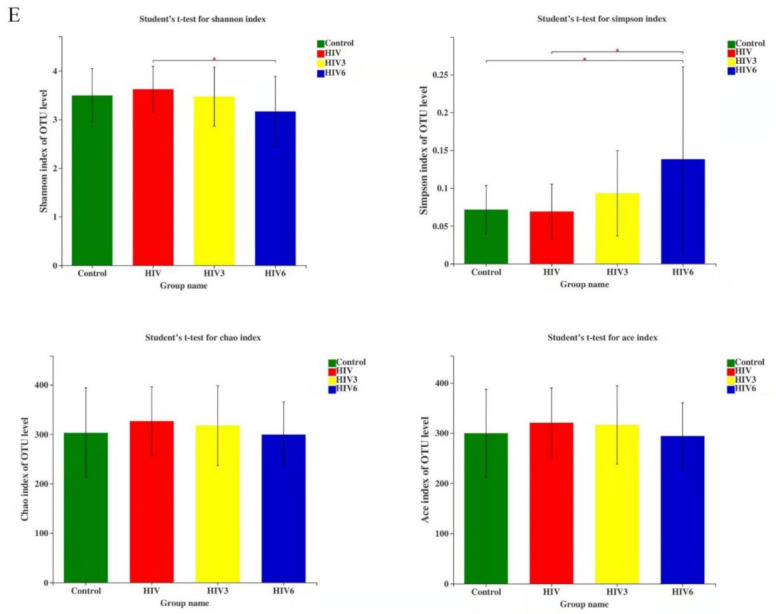
Comparison of salivary microbiome structure among healthy controls and HIV infections, ART at three and six months. (**A**) Rarefaction curves of the bacterial operational taxonomic units (OTUs) derived from the four groups. (**B**) The Venn diagram illustrates the shared and independent OTUs in the salivary microbiome among the groups. (**C**) The community barplot showed the microbial community structure among the saliva samples. (**D**) Principal coordinate analysis of the microbiota based on the Bray–Curtis. (**E**) The Shannon, Simpson, Chao and Ace indices were used to estimate the microbial alpha diversity. HIV3: HIV infections group three months after ART; HIV6: HIV infections group six months after ART. * 0.01 < *p* ≤ 0.05.

A total of 3,440,992 high-quality sequences was generated after quality checks. From healthy controls and HIV infections (before and over the course of ART), 919,503 and 2,521,489 sequences were obtained, respectively. Rarefaction curves showed a great sequencing depth of the saliva samples in our study that was suitable for the downstream phylogenetic analysis (Figure 1A). Taxonomic analysis illuminated 31 phyla, 56 classes, 113 orders, 189 families, 451 genera, and 797 species, and a total of 1177 OTUs were identified from the filtered sequences, specifically 835 OTUs in healthy controls, 824 OTUs in HIV infections at baseline, and 800 and 695 OTUs in the ART-treated group following three and six months of ART, respectively. Additionally, 471 OTUs were shared by the four groups. The shared OTUs among the groups are displayed in a Venn diagram (Figure 1B).

**Figure 2:**
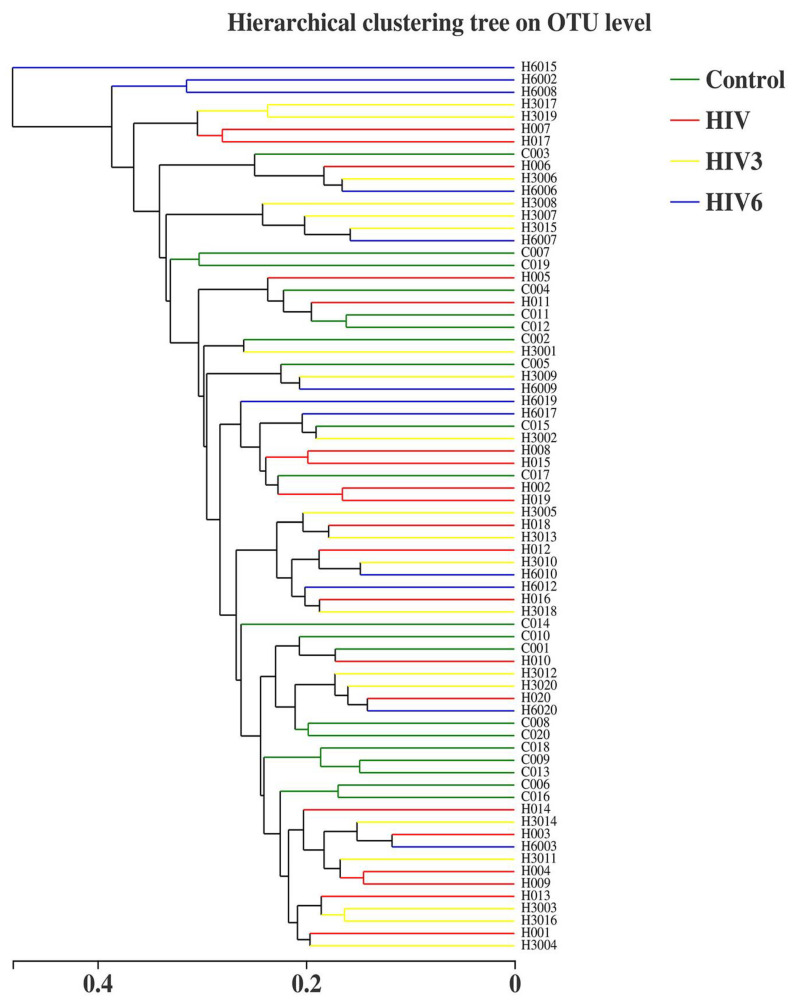
Hierarchical cluster analyses based on the OTU level; both the HIV infections group and healthy controls in our study exhibited a higher degree.

Based on the results of taxonomic analysis, similar microbial community structures among the saliva samples were found to be dominated by the following detected bacterial phyla: *Firmicutes*, *Proteobacteria*, *Bacteroidetes* and *Actinobacteria* predominated, followed by *Fusobacteria* and *Saccharibacteria* (Figure 1C). These six predominant bacterial phyla constituted over 98% of the total salivary microbiota in the healthy controls and the HIV infections. To compare the bacterial diversity among different samples, the diversity indices (Shannon and Simpson) and richness estimators (ACE and Chao) were used for microbial alpha diversity analysis. Although the variations were different among the groups, differences were not statistically significant between the HIV-infected patients at baseline and healthy controls, while the Shannon index was lower and the Simpson index was higher in HIV-infected patients after six months of ART (Figure 1E). These data indicated that the alpha diversity of the salivary microbiota in HIV infections before ART did not show differences from the healthy controls, but became lower after ART. Likewise, no differences were found in the salivary microbial communities within the four groups based on the principal coordinate analysis (PCOA), which did not produce a conspicuous separation between the different saliva groups (Figure 1D). Nonetheless, taking the HIV infections together, all of the HIV infections and healthy controls analyzed in our study exhibited a higher degree of clustering in terms of the microbial community composition, as shown by the hierarchical cluster analyses (Figure 2).

### 2.3. Different Salivary Microbiome between HIV Infections before ART and HIV-Uninfected Group

**Figure 3:**
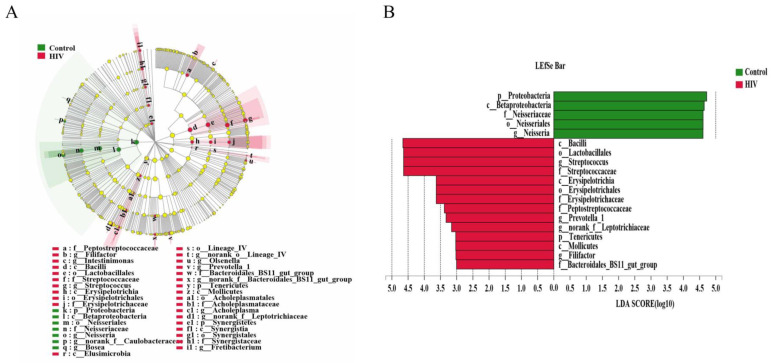

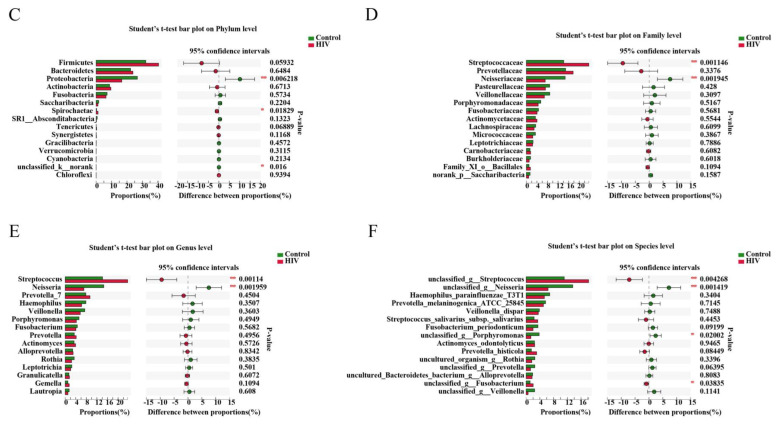
Different salivary microbiomes between HIV infections before ART and healthy controls. LEfSe identified the taxa with the greatest differences in abundance between healthy controls and HIV infections, healthy control-enriched taxa (green) and HIV-infected-enriched taxa (Red). Only the taxa meeting a significant LDA threshold value of >3 were shown (**A**,**B**). Comparisons of the relative abundance at the levels of bacterial phylum (**C**), family (**D**), genus (**E**) and species (**F**) between healthy controls and HIV infections. * 0.01 < *p* ≤ 0.05, ** 0.001 < *p* ≤ 0.01.

Although the differences in bacterial diversity were not obvious, the specific enriched bacterial taxa in the HIV-infected group before ART and the HIV-uninfected group were identified by linear discriminant analysis effect size (LEfSe). A cladogram was constructed to describe the structure of the salivary microbiota and the different predominant bacterial taxa between the two microbial communities. In addition, alterations in the composition of the salivary microbiota between the healthy controls and HIV-infected group before ART were also explored, utilizing the Student’s *t*-test at multiple taxonomic levels. LEfSe demonstrated that *Neisseria* was enriched in the healthy controls, whereas *Streptococcus*, *Prevotella_1*, nornank*_f_Leptotrichiaceae* and *Filifactor* were enriched in HIV-infected patients before ART (Figure 3A,B), which might be referred to as salivary biomarkers to distinguish the individuals with HIV infections. Furthermore, at the phylum level, there was a significantly increased abundance of *Spirochaetae* and a decreased abundance of *Proteobacteria* in the salivary microbiome of the ART-naïve, HIV-infected group, compared with the HIV-uninfected group (Figure 3C). At the family level, we found that *Neisseriaceae* was more prevalent in the HIV-uninfected group, whereas the relative abundance of *Streptococcaceae* in HIV infections of the ART-naive group was significantly higher (Figure 3D). At the genus level, two predominant genera of the HIV-uninfected group and of the ART-naïve, HIV-infected patients were different. *Streptococcus* was present at a higher relative abundance in the HIV-infected group, while *Neisseria* was found in the healthy controls (Figure 3E). All of these alterations indicated dysbiosis of the salivary microbiome after infection with HIV.

### 2.4. Impact of Three and Six Months of ART on the Salivary Microbiome in HIV Infections

**Figure 4:**
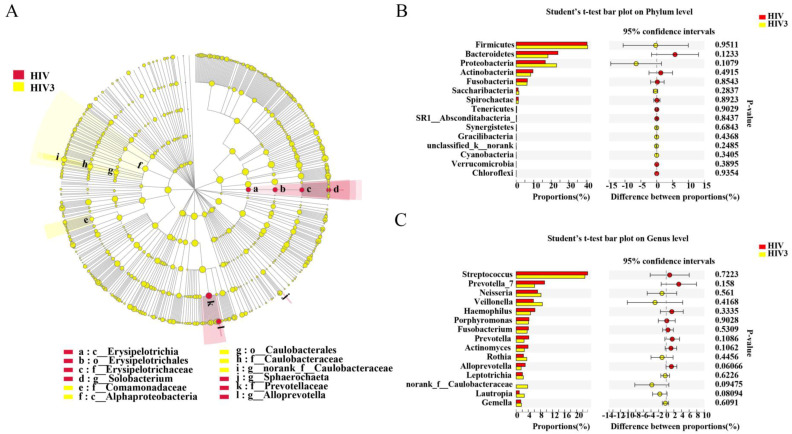

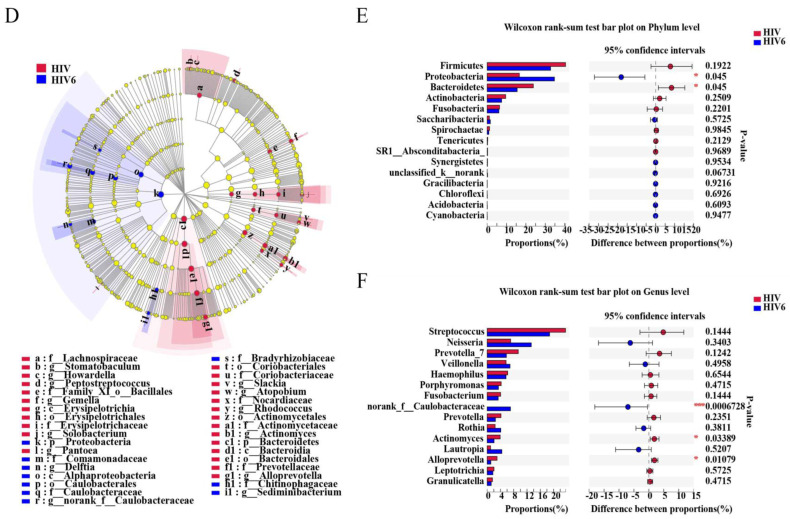
Impact of three and six months of ART on the salivary microbiome in HIV-infected patients. Comparisons of salivary microbiome between HIV before ART and HIV at three months (**A**–**C**) and six months (**D**–**F**) after ART. * 0.01 < *p* ≤ 0.05, *** *p* ≤ 0.001.

**Figure 5:**
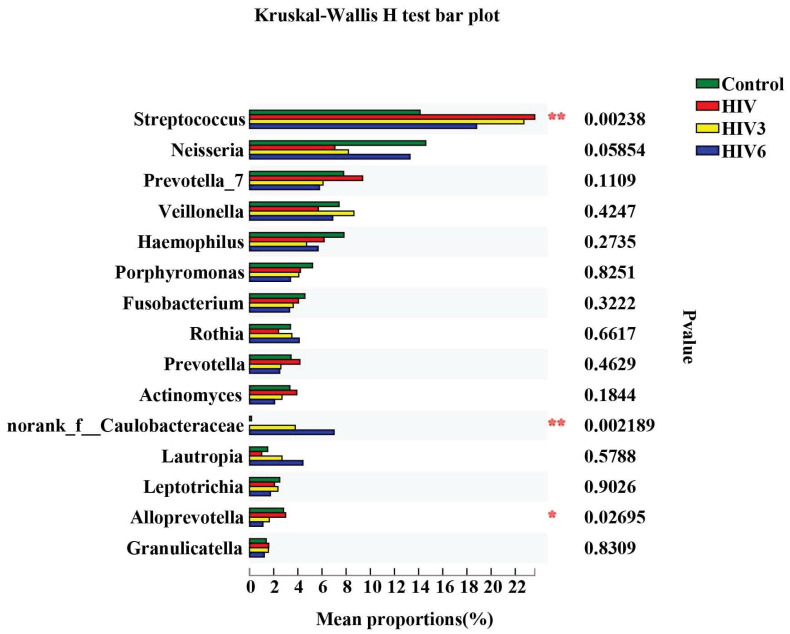
Comparative analysis of the four groups at the genus level. The tope 15 genera were picked out and analyzed by Kruskal–Wallis H test. * 0.01 < *p* ≤ 0.05, ** 0.001 < *p* ≤ 0.01.

All of the subjects infected with HIV in our study received ART voluntarily. However, only 12 HIV-infected patients were available at the six months timepoint. Two patients moved to another location, four patients stopped ART due to the side effects, and two patients were not willing to continue their participation in the study. To evaluate the influence of ART on the salivary microbiome in HIV infections, a longitudinal study was applied to explore the salivary microbiome at three and six months after the initiation of ART. In our comparisons of the overall salivary microbial diversity among the HIV infections before ART and three and six months after ART, the alpha diversity was reduced after six months of ART (Figure 1A,B). Similarly, we noted that ART impacted the composition of the salivary microbiome of the HIV infections in the early stage by using the LEfSe and comparative analyses. After three months of ART, we found that *Solobacterium*, *Sphaerochaeta* and *Alloprevotella* were enriched in the HIV group before ART, while *nornank_f_Caulobacteraceae* was enriched after ART for three months. However, a comparative analysis did not detect this difference after three months of ART. After six months of ART, 34 discriminative microbiota were revealed, including the *Proteobacteria*, *Bacteroidetesa* and the *nornank_f_Caulobacteraceae*, *Actinomyces*, and *Alloprevotella* (Figure 4D–F). We also observed that the microbiome became more similar to the microbiome of the healthy controls after ART, although a difference remained among the groups (Figure 5).

### 2.5. Associations of the Salivary Microbiome with Demographic Characteristics, CD4+ T cell Count, and VL

**Figure 6:**
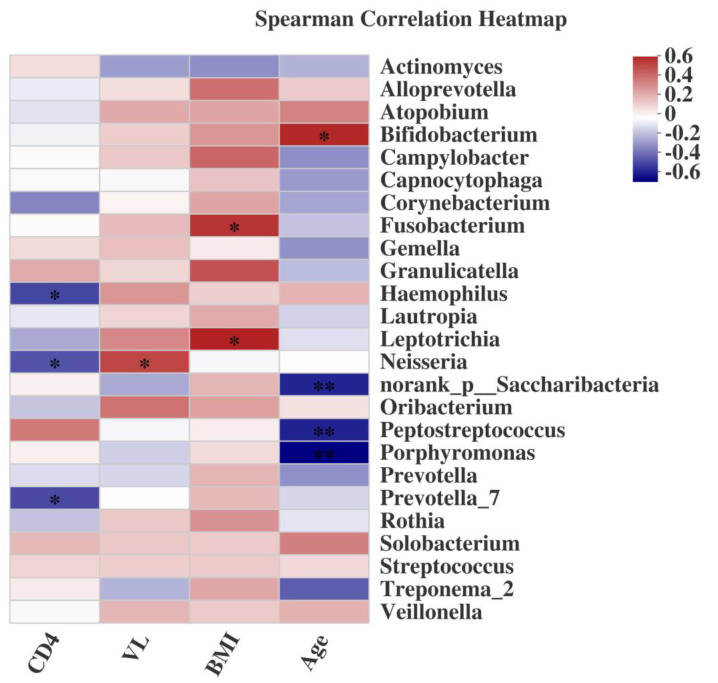
Spearman’s correlation heatmap of salivary microbiome (the abundance of the top 25 genera) with demographic characteristics, CD4+ T cell count, and VL. Positive correlations were shown in red and negative correlations in blue. * 0.01 < *p* ≤ 0.05, ** 0.001 < *p* ≤ 0.01.

Additionally, focusing on blood parameters we found that the peripheral CD4+ T cell count correlated negatively with the abundance profiles of *Provotella_7*, *Neisseria* and *Haemophilus* (Figure 6). We also detected potential relationships between salivary microbiome profiles and plasma HIV RNA levels. *Neisseria* abundance correlated positively with VL (Figure 6). Intriguingly, this pattern directly contrasted with the negative correlation observed between the same genera and circulating CD4+ T cell count. On the other hand, the genera *Fusobacterium* and *Leptotrichia* were correlated positively with the BMI (Figure 6). However, a negative correlation of *Bifidobacterium* and a positive correlation of *Peptostreptococcus* and *Porphyromonas* with age were founded.

## 3. Discussion

Young MSM living with HIV are at particularly high risk given the Chinese HIV epidemic. They accounted for 25.5% of the country’s newly identified HIV cases and AIDS patients in 2017, and HIV infections in MSM in China are expected to continue to expand in the absence of early and easy HIV testing [29]. Taking advantage of the noninvasive, convenient and unsuspicious features of saliva sampling, and the development of high-throughput sequencing technology, we explored the salivary microbiome in HIV-infected patients who were MSM. In the longitudinal study, we observed shifts in the abundance of the *Streptococcus*, *Neisseria*, *Prevotella_1* and *Alloprevotella*. More importantly, our study bridged correlations between the salivary microbiome and the CD4+ T cell count and VL, which provide an alternative sample to the invasive blood test for HIV infections.

The human oral microbiome is a complex multi-microbial community in delicate balance [30]. Alterations of the salivary microbiota profiles have been demonstrated with HIV infections in several previous cross-sectional studies [24,25,26], which revealed that oral dysbiosis driven by enriching or reducing certain taxa is significantly associated with HIV infection. Because the microbiota alterations in HIV infections are affected by multiple elements (population, sex, age, sample type, oral hygiene, and ART) [31,32], the alterations of the oral microbial community’s structure are not always consistent with each other. Li Y [23] considered that it was likely that an increased proportion of opportunistic microbes leads to a decrease in microbial diversity in HIV-positive samples, and diversity was reduced following highly active antiretroviral therapy (HAART). The role that the salivary flow plays in shaping the organization of microbial communities in human health has been documented [33], and evidence has shown that the salivary flow was reduced after HAART [34], which in turn might affect the richness and composition of the salivary microbiota. In the present study, there was no noticeable difference in microbial diversity between the healthy controls and the HIV-infected patients before ART and three months after ART, consistent with the previous findings in the saliva and plaque [12,35]. All of these observations revealed that it is the dysbiosis (microbial imbalance) rather than the presence or absence of specific microbes that is more closely associated with HIV infections. However, we found that the alpha diversity was reduced after six months, which may have been due to the use of ART.

Assisted by the development of sequencing techniques, the researchers have begun to shift the study of disease from pathogens to pathobiomes, the aim being a comprehensive understanding of the microbial ecosystem. In recent years, the role of the microbiome in HIV infections has been explored extensively. Although the alpha diversity differs among studies, most previous research has detected alterations of microbial composition in HIV infections [35,36,37]. In our study, we found a significantly increased abundance of *Spirochaetae* and a decreased abundance of *Proteobacteria* in the salivary microbiome of HIV-infected patients. These results are consistent with some studies and contrary to others. For example, in the gut and the lingual, *Proteobacteria* were increased, in the palatine tonsil *Proteobacteria* decreased, while in the rectum there was no difference in this phylum [30,38,39,40]. One possible explanation is that every organ or sample has a specific microbiome with its own characteristics that has different responses to an HIV infection. What is more, the dysbiosis of normal microbiota may influence HIV transmission, prevention, progression and prognosis [38]. In the analysis of the salivary microbiome in HIV-infected patients, the LEfSe analysis showed that the *Neisseria* were enriched in the healthy controls, whereas *Streptococcus*, *Prevotella_1* and *Filifactor* were enriched in HIV infections before ART. However, a study by Mukherjee PK et al. [27] found that *Streptococcus*, *Prevotella* and *Neisseria* are the oral core bacteriome of both HIV-uninfected and HIV-infected patients. Zhang F et al. [41] found that *Streptococcus* and *Neisseria* were involved in the development of AIDS with different periodontitis, and that *Neisseria* was always enriched in HIV infections regardless of sampling site and probing depth level. In recent years, as a newly recognized periodontitis-associated pathogen, *Filifactor* has exhibited expected associations with periodontitis in perinatally HIV-exposed uninfected youth, associations not observed in perinatally HIV-infected youth [42].

In the longitudinal study, we followed HIV-infected patients for three and six months after ART. At the early stage of the three-month period after ART, we did not find any difference compared with HIV-infected patients before ART. After six months, the HIV-infected patients experienced an increased CD4+ T cell count and a decreased VL. Furthermore, comparing the salivary microbiota between the six-month ART group and HIV-infected patients at baseline, we found a reversal of the frequencies of *Streptococcus*, *Neisseria*, *Actinomyces*, and *Alloprevotella*, although the salivary microbiota were not completely restored when compared with the healthy controls. After ART, immune function in the HIV-infected patients was reconstructed, and the microbial dysbiosis was attenuated. However, recent studies have reported that CD4+ T cell counts and immune status have no effect on the oral, airway or palatine tonsil microbiome in HIV-infected individuals [24,38]. Another study showed that shifts in the oral microbiome after ART were influenced by the immune status [35]. Collectively, we considered that although ART recovered the immune status of HIV infections, the microbiome community’s composition could not be restored to that of the healthy microbiome, suggesting that the HIV infection itself makes a difference to the microbiome.

By association analysis of the salivary microbiome with demographic characteristics, CD4+ T cell count and VL, we found that certain microbiomes were related to these parameters. Interestingly, we found that many microbial taxa were positively correlated with the BMI, which usually is thought to be closely related to the gut microbiome [43]. We also detected associations between age and microbiome, contrary to a previous report [35]. However, a study of HIV-infected women of different ages found that the salivary microbiome is associated with aging [44]. Our results suggest that a salivary microbiome test could serve as a biomarker for the prognosis of HIV infections following ART.

Although in this study we report the altered salivary microbiome of healthy controls, and the HIV infections at baseline and following three and six months of ART using individuals as their own controls, certain limitations exist in our study. The lack of HIV-negative MSM controls in our study is one limitation. Given significant individual variability, HIV-uninfected MSM individuals are necessary controls for distinguishing the effects of HIV infection itself on the composition of the microbiome, especially in China where the majority of HIV infections are MSM. However, it is hard for us to contact the HIV-uninfected MSM before they come to a voluntary visit for the reason that MSM is private and furtive in China. The second limitation is that only HIV infections undergoing six months of ART were followed up in this study. To better understand the relationship between the salivary microbiome and the immune status in HIV infections, an investigation of long-term ART is needed in future studies. Finally, HIV-infected patients without ART were not followed in our longitudinal study, so we could not decide whether there is a direct impact of the ART itself on the microbiome [23,27].

## 4. Materials and Methods

### 4.1. Study Subjects

Twenty HIV-infected patients before ART were randomly recruited between April 2017 and June 2018 from patients newly admitted to the Wuhan Medical Treatment Center, Wuhan, China. Twenty age- and gender-paired HIV-uninfected healthy Chinese individuals were voluntarily recruited from the students and employees of Wuhan University, China. The demographic data (for all subjects) and medical data (for HIV infections) included gender, age, body mass index (BMI), VL, CD4+ T cell counts, initial type and date of ART medications; data collection and evaluation were carried out before and at three and six months after initiation of ART.

The inclusion criteria were as follows: (1) patients with HIV infection (diagnosed within one year) not yet undergoing ART; (2) over 18 years of age; (3) CD4+ T cell counts ≥ 200 cells/μL.

Exclusion criteria were as follows: (1) systematic diseases such as hypertension, diabetes mellitus, and autoimmune diseases; (2) intake of antibiotics or non-steroidal anti-inflammatory drugs within three months before ART; (3) HIV infections with obvious clinical oral symptoms (caries, periodontal disease, mucous disease); (4) accompanied by other infectious diseases, such as syphilis, HBV/HCV or tuberculosis.

### 4.2. Sample Collection and Initial Processing

Before the sample collection, a comprehensive standardized oral examination was conducted by one of two clinical dentists who have undergone a conformance test. Unstimulated whole saliva samples were collected between 9:00 AM and 11:00 AM in this study, if possible. All subjects were required not to brush their teeth, or eat or drink anything for at least 2 h before saliva sample collection. After 5 min of rest without talking, a saliva DNA collector (DNAgard^®^ Saliva, Boykyo Pharmaceutical Co. Ltd., Tokyo, Japan) was slightly affixed to the underlip to collect 2 mL saliva sample according to the instructions. Saliva samples were quickly transported on ice and stored at −80 °C in the laboratory before further application.

### 4.3. Genomic DNA Isolation and PCR Amplification

Bacterial DNA from saliva samples was extracted using the FastDNA^®^ Spin Kit for Soil (MP Biomedicals, Solon, OH, USA) according to manufacturer’s protocols. The total bacterial DNA concentration and purification were measured by a NanoDrop 2000 UV-vis spectrophotometer (Thermo Scientific, Wilmington, NC, USA), and 1% agarose gel electrophoresis was used to check DNA quality. All genomic DNA samples were stored at −80 °C before further use.

The genomic DNA was PCR amplified using bacteria 16S rRNA gene primers targeting the V3–V4 hypervariable regions: primers (forward) 338F (5′-ACTCCTACGGGAGGCAGCAG-3′) and (reverse) 806R (5′-GGACTACHVGGGTWTCTAAT-3′). In total, 10 ng of template DNA, 4 μL of 5 × FastPfu Buffer, 2 μL of 2.5 mM dNTPs, 0.8 μL of each primer (5 μM), and 0.4 μL of FastPfu Polymerase were mixed in triplicate for a 20 μL PCR mixture. The PCR reaction procedures and cycling conditions were carried out using the following protocol: denaturation at 95 °C for 3 min, 29 cycles of denaturation at 95 °C for 3 s, annealing at 55 °C for 30 s, elongation at 72 °C for 45 s, and a final extension step at 72 °C for 10 min. PCR reactions were performed by a thermocycler PCR system (ABI GeneAmp 9700, Grand Isand, NY, USA). The resulting PCR products were extracted by a 2% agarose gel, and were then purified using the AxyPrep DNA Gel Extraction Kit (Axygen Biosciences, Union City, CA, USA), and were quantified using QuantiFluor™-ST (Promega, Madison, WI, USA).

### 4.4. Illumina MiSeq Sequencing

After being purified, the PCR amplicons from each library were pooled in equimolar and run on an Illumina MiSeq platform (Illumina, San Diego, CA, USA), and then paired-end sequenced (2 × 300) according to the standard protocols by Majorbio Bio-Pharm Technology Co. Ltd. (Shanghai, China).

### 4.5. Data Processing and Sequence Analysis

The datasets generated and/or analyzed during the current study are available in a repository at the NCBI Sequence Read Archive. BioProject number: PRJNA566246; Accessing number: SRP222739.

Raw fastq files were demultiplexed and quality filtered by Trimmomatic, and then merged by FLASH according to the following criteria: (i) Reads at any site with an average quality score < 20 over a 50 bp sliding window were truncated. (ii) Samples were distinguished according to barcode and primer at both ends of the sequence. Exactly matched barcodes and primers less than 2 nucleotide mismatching were allowed, and reads containing ambiguous bases were removed. (iii) According to overlap relation between PE reads, pairs of reads were merged into a sequence, and the minimum overlap length was 10 bp.

Operational taxonomic units (OTUs) were clustered for non-repeating 16S rRNA gene sequences with 97% similarity cutoff using UPARSE (version 7.1 http://drive5.com/uparse/), and chimeric sequences were identified and removed using UCHIME. The taxonomy of each OTU representative sequence was analyzed by the Ribosomal Database Project (RDP) Classifier algorithm (http://rdp.cme.msu.edu/) against the Silva (SSU123) 16S rRNA database using a confidence threshold of 70%.

The OTUs were used for alpha diversity indices (Shannon, Simpson) and richness estimators (ACE and Chao1) using mothur [45]. The microbial community barplots, Venn diagrams, rarefaction curve analyses, and heatmaps were generated based on the relative abundance of OTUs using the R statistical software package.

The phylogenetic beta diversity for comparative analysis among different samples was calculated using QIIME (http://qiime.org/scripts/assign_taxonomy.html) [46], and rendered by the R statistical software package. Hierarchical cluster analysis was used to analyze the similarities of the microbial communities of all individuals at the OUT level. Permutational multivariate analyses of variance of the distance matrices between groups were compared using the R statistical software package. FDR (false discovery rate) was applied in the comparisons of differences between groups. PCOA was performed at the OUT level based on Bray–Curtis. Linear discriminant analysis effect size (LEfSe) was utilized to identify statistically different bacterial taxa in their relative abundances between HIV positive young men and healthy controls. Only the taxa meeting a significant LDA threshold value of > 3 were shown.

### 4.6. Statistical Analysis

Alpha diversities and the relative abundances between two groups were compared using Student’s *t*-test and Wilcoxon rank-sum test. A Spearman’s Rank correlation coefficient test was applied to evaluate the correlation between the salivary microbiome and demographic characteristics (BMI and age), CD4+ T cell count and VL. *p* < 0.05 was considered statistically significant.

### 4.7. Ethics Statement

The human subject protocol for all procedures was approved by the Ethics Committee of the School and Hospital of Stomatology, Wuhan University and the Wuhan Medical Treatment Center, Wuhan, China. Written informed consent was signed by all participants. 

## 5. Conclusions

Based on our data, we conclude that salivary microbiome dysbiosis occurs in the early stage of HIV infection among a population of young Chinese men, and we identified several altered microbial taxa that represent the salivary dysbiosis. Furthermore, certain salivary microbiota, such as *Streptococcus*, *Prevotella_1*, and *Filifactor*, are potential salivary bacterial biomarkers for early and easy HIV testing, and *Neisseria* could be an optional biomarker for use in the prognosis of HIV infection because of its advantages and rapidly decreasing sequencing costs. Ultimately, further microbiome sequencing and longitudinal studies with larger sample sizes are necessary in order to better and more accurately discriminate saliva samples, and eventually turn this approach into a clinical application.

## Figures and Tables

**Table 1:** Demographic and clinical parameters of the HIV-infected patients at baseline and six months after ART and healthy controls.

	HIV	HIV6	Control
Gender			
Men	20	12	20
Women	0	0	0
Age (mean ± SD)	26.30 ± 5.41		26.30 ± 5.41
BMI (mean ± SD)	21.31 ± 3.19		22.31 ± 3.04
Transmission			
Injection Drug Use	0	0	0
Heterosexual	1	0	0
Homosexual	19	12	0
CD4+ T cell count (Range) (cells/μL)	219–652	323–798	/
CD4 ≥ 500 cells/μL	3	10	/
CD4 < 500 cells/μL	17	2	/
Viral Load (copies/mL) (Range)	5059–8,749,628	TND–1930	/

Abbreviations: HIV—HIV infections group before ART. HIV6—HIV infections group six month after ART. BMI—body mass index; SD—standard deviation; TND—target not detected.
