# Correction: Li et al. Altered Salivary Microbiome in the Early Stage of HIV Infections among Young Chinese Men Who Have Sex with Men (MSM). *Pathogens* 2020, *9*, 960

**DOI:** 10.3390/pathogens11070785

**Published:** 2022-07-11

**Authors:** Jin Li, Shenghua Chang, Haiying Guo, Yaoting Ji, Han Jiang, Lianguo Ruan, Minquan Du

**Affiliations:** 1 The State Key Laboratory Breeding Base of Basic Science of Stomatology (Hubei-MOST) & Key Laboratory of Oral Biomedicine Ministry of Education, School & Hospital of Stomatology, Wuhan University, Wuhan 430079, China; lijin891212@whu.edu.cn (J.L.); changshh2017@whu.edu.cn (S.C.); haiyingguo@whu.edu.cn (H.G.); yaotingji@whu.edu.cn (Y.J.); jianghan@whu.edu.cn (H.J.); 2 Department of Infectious Diseases, Jin Yin-tan Hospital, Wuhan 430023, China

In the original publication [1], there was a mistake in Figure 1E as published. We had placed two histograms about “Simpson index” instead of “Shannon and Simpson” index. The corrected Figure 1E appears below.


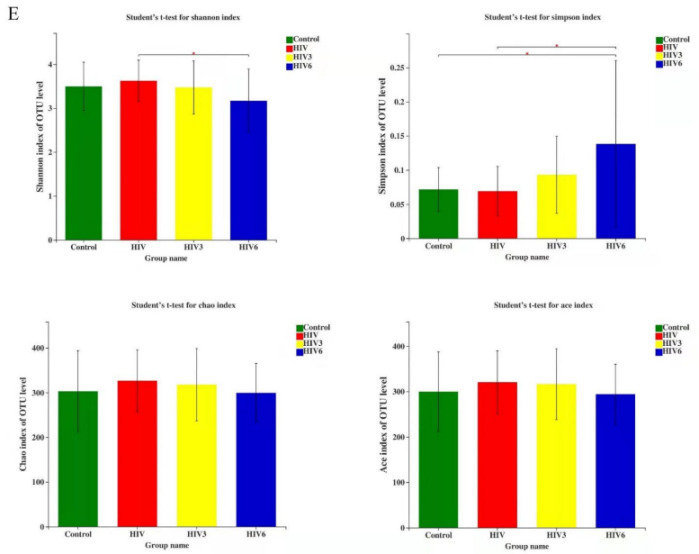


The authors apologize for any inconvenience caused and state that the scientific conclusions are unaffected. The original publication has also been updated.
